# Estimation of footprints of the canine stifle ligaments using deformable shape templates of bones

**DOI:** 10.1038/s41598-024-55116-3

**Published:** 2024-02-26

**Authors:** Yu-Ying Lin, Cheng-Chung Lin, Ching-Ho Wu

**Affiliations:** 1 Institute of Veterinary Clinical Sciences, National Taiwan University, Taipei, Taiwan; 2 Department of Electrical Engineering, Fu Jen Catholic University, New Taipei City, Taiwan

**Keywords:** Biotechnology, Computational biology and bioinformatics, Anatomy, Engineering, Mathematics and computing

## Abstract

Knowledge regarding the ligament footprints in the canine stifle is essential for biomechanical modeling of the joint and patient-specific surgical planning for anatomical ligament reconstruction. The present study aimed to establish and evaluate deformable shape templates (DSTs) of the femur and tibia with footprints of the cruciate and collateral ligaments embedded for the noninvasive estimation of ligament footprint positions. To this end, a data set of computed tomography (CT)-derived surface models of the femur and tibia were established and used to build statistical shape models (SSMs). The contours of the stifle ligaments were obtained from CT scans of 27 hindlimb specimens with radio-opaque markings on the ligament footprints. The DST, constructed by embedding averaged footprint contours into the SSM, was used to estimate subject-specific ligament footprints in a leave-one-out cross-validation framework. The DST predictions were compared with those derived from radio-opaque-marked footprints. The results showed that the averaged Euclidean distances between the estimated and reference footprint centroids were less than 1.2 mm for the cruciate ligaments and 2.0 mm for the collateral ligaments. The DST appeared to provide a feasible alternative approach for noninvasively estimating the footprints of the stifle ligaments in vivo.

## Introduction

Femorotibial ligaments, including cruciate and collateral ligaments, play important roles in stabilizing the stifle joint in dogs, with their anatomical locations and biomechanical functions well-documented^1^. In particular, the cranial cruciate ligament (CrCL) in dogs has been widely studied, as its disorders are the most common causes of canine hindlimb lameness^2^. A quantitative description of subject-specific attachment sites of the ligaments was fundamental in the biomechanical modeling of the stifle^3,4^ in relevant biomechanical studies and identification of the ligament footprint location for anatomical intraarticular ligament reconstruction^5^.

To attain accurate computer modeling of the stifle joint, it is essential to obtain coordinates of the ligament endpoint locations. These coordinates were previously obtained from a computed tomography (CT) scan of a canine cadaver^4^ or referenced literature sources^3^. A musculoskeletal computer model incorporating generic ligament information may yield less realistic simulation outcomes, thereby compromising the accuracy of subject-specific analysis in joint biomechanics. On the other hand, preceding studies have indicated that anatomical placement of the bone tunnel may lead to a better clinical outcome of intra-articular CrCL repair^2,6,7^. In previous studies, it had been shown that the use of arthroscopy, coupled with adjustable aiming devices, effectively reduced tunnel misplacement in medium to large-sized dogs^6^. Nonetheless, the arthroscopic techniques appeared to be less applicable for smaller joint spaces in smaller dogs or those affected by osteoarthritis. In scenarios where arthroscopy is not utilized, it is also challenging to directly observe the CrCL footprint on the canine stifle intraoperatively, especially at the femoral site. Therefore, a method enabling the assessment of CrCL footprint locations may fulfill the requirements of biomechanical studies and clinical demands.

Various medical image-based methods have been proposed to describe or predict the ligament footprint locations of the human knee, namely, arthroscopy^8^, radiography^9,10^, magnetic resonance (MR) imaging^11,12^, and deformable bone templates^13,14^. Arthroscopy allows direct visualization of footprint sites during the operation but may not be suitable for application in preoperative planning or biomechanical research. The radiographic method is limited to two-dimensional (2D) descriptions; as a result, any improper positioning of patient poses can lead to measurement errors. While the MR images provide complete volumetric information on the bone and soft tissues of the stifle, the process of manually marking ligament insertion sites can lead to considerable intra- and interobserver differences^12^. Model-based prediction of ligament attachment sites allowed the elimination of observer variability. To this end, Ascani et al. introduced a reference registration atlas, wherein bony landmarks and ligament endpoints were identified from CT and MR images, respectively. This atlas was then used to predict subject-specific ligament origins in the human knee through an affine transformation^13^. Similarly, Pillet et al. developed a bone template embedding with predefined ligament endpoints from cadavers to provide an estimation of subject-specific ligament origins in the human knee through a shape transform process^14^.

To date, the radiographic measurement of femoral CrCL footprint locations in dogs has been documented in one study^15^. Quantitative three-dimensional (3D) descriptions of canine ligament footprints are still lacking. There is currently no objective prediction method available for identifying ligament footprints in dogs. Therefore, the objectives of the present study are threefold: first, to establish a deformable shape template (DST) of the femur and tibia embedded with the footprint contours of the CrCL, caudal cruciate ligament (CaCL), lateral collateral ligament (LCL), and medial collateral ligament (MCL) based on the statistical shape modeling (SSM) technique for describing and estimating ligament footprints; second, to analyze the 3D dispersions of the ligament footprints based on the DST; and third, to evaluate the performance of the DST in predicting the subject-specific stifle ligament footprints.

## Methods

### Subjects

To obtain stifle ligament information, 27 femur and tibia (Right: 14; Left: 13) specimens were harvested from cadaveric hindlimbs of 16 adult Taiwan dogs with an average body weight of 15.6 ± 4.3 kg. The cadaveric hindlimbs utilized in this study were sourced from a prior research project ^16^ (IACUC number: NTU-109-EL-00070) conducted at National Taiwan University Veterinary Hospital and repurposed to fulfill the objectives of the current experiment. All specimens underwent postmortem orthopedic and radiographic examination of the hindlimb joints. The included limbs were disarticulated from the coxofemoral joints and then stored in a − 20 °C refrigerator with saline-soak towel wrapping before processing.

To build statistical shape models (SSMs) for the femur and tibia, the training dataset was created by retrospectively collecting CT data from our former studies^17–19^. This dataset comprised CT data from a total of 40 dogs, with an average body weight of 22.7 ± 4.6 kg and an age of 3.6 ± 1.7 years. The cohort was composed of 27 Taiwan dogs, 11 Labradors, and one German Shepherd and Golden Retriever.

### Identification of the ligament footprints

**Figure 1:**
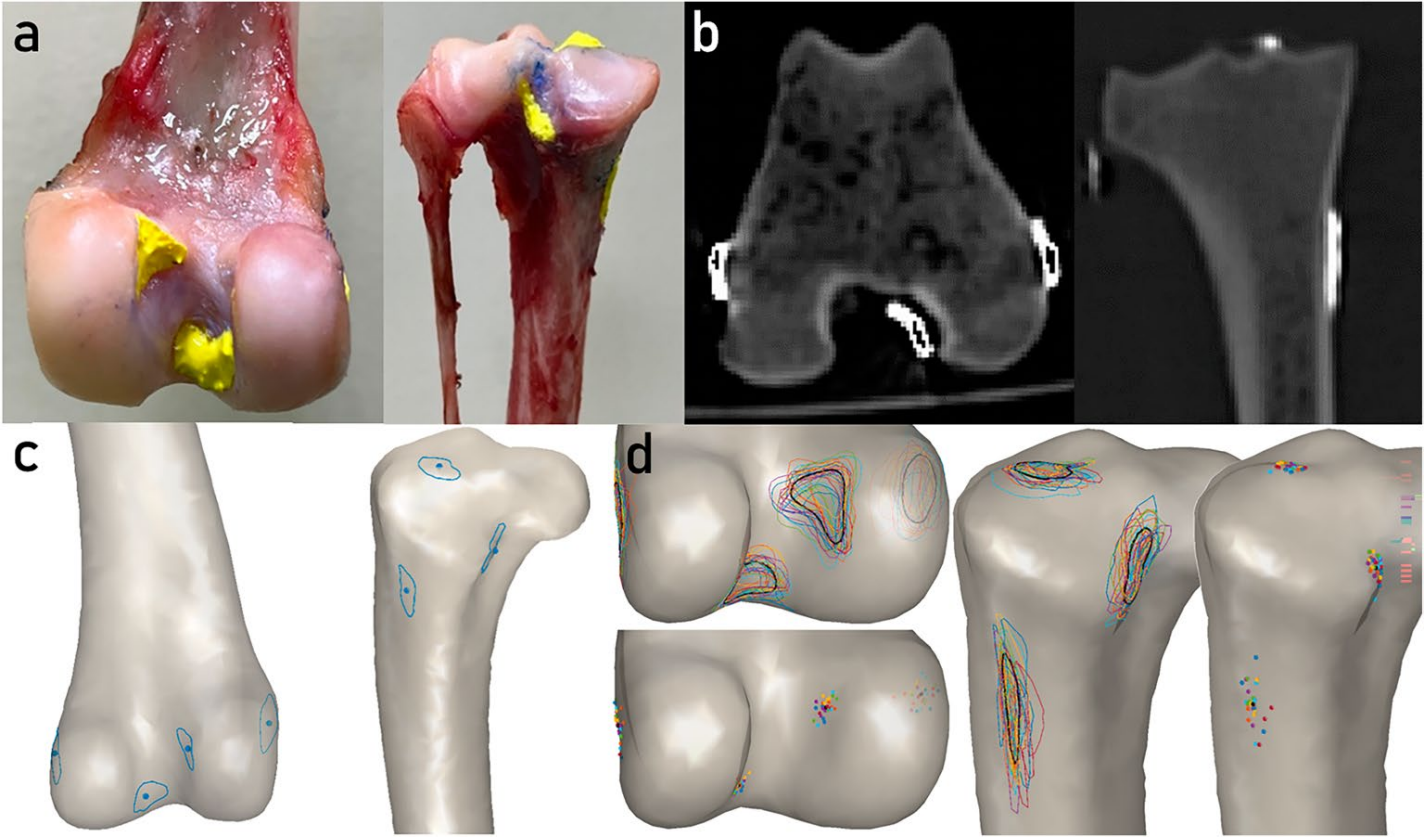
The process for the identification of ligament footprints and the creation of deformable shape templates of bones. (**a**) The stifle ligament footprints on the bone were marked with radio-opaque paint (yellow marks). (**b**) CT images of the femur and tibia with radio-opaque paint marked on the ligament footprints (high-intensity regions). (**c**) The contours (blue outline) and centroids (blue dots) of the ligament footprints on the surface models of the femur and tibia. (**d**) The mean shape of the SSMs of the femur and tibia embedding contours (colored outlines) and centroids (colored dots) of ligament footprints from all dogs. Additionally, the averaged contours and centroids of the ligament footprints are depicted in black.

Each limb specimen was fully dissected following the steps described below. First, the skin, muscles, and patella were removed. Second, the four stifle ligaments were carefully separated from the surrounding soft tissue. Third, the CrCL, CaCL, MCL, and LCL were removed by cutting at their insertion site. After dissection, a radio-opaque paint made of acrylic paint mixed with barium sulfate was used to mark the ligament footprint areas (Fig. 1a). All procedures mentioned above were performed by one operator (YYL).

The femur and tibia with radio-opaque paints were scanned by CT (Activiom 16; Toshiba, Japan), with which transverse image slices of each femur and tibia were obtained (Fig. 1b) with a resolution of 512 × 512 pixels and pixel sizes ranging from 0.346 to 0.568 mm. The interslice distance was standardized at 0.3 mm. The regions of the femur, tibia, and seven ligament footprints (i.e., CrCL, CaCL, and MCL at both femoral and tibial sites and LCL at the femoral site) were identified and semiautomatically segmented based on the region-growing algorithm. Given the segmented regions of individual bones and footprints, the corresponding polygonal surface models were created and exported in Stereolithography format using a custom-made program in MATLAB (R2020a, MathWorks Inc., Natick, MA).

### Statistical shape model (SSM) of the bones

The SSMs of the femur and tibia were created following the steps described in a preceding study^20^. First, shape models of the bilateral femur and tibia were reconstructed with the respective CT images. A total of 40 right femoral, 39 left femoral, 28 right tibial, and 26 left tibial shape models were created. Any bones with partial regions extending beyond the CT field of view were excluded from the construction SSMs. The right femoral and tibial shape models were mirrored to the left side, and vice versa, which eventually yielded a total of 79 bilateral femur shape models and 54 bilateral tibia shape models.

A reference shape mesh was randomly selected from the shape models of the femur and underwent resampling processing, yielding a uniformly distributed set of 3000 nodes. Second, the reference mesh was transformed to match each bone shape model sequentially using the Coherent Point Drift^21^ to establish a consistent number of corresponding nodes among all bone shape models. Third, generalized Procrustes analysis was performed to best align all shape models. Fourth, the mean shape was obtained by taking an average over aligned bone shapes, and the principal modes of shape variation were identified using principal component analysis. The mean shape and shape variations form the basis of SSM. The same procedure was repeated to establish an SSM of the tibia.

### Deformable shape template (DST)

A ligament footprint contour estimation approach, referred to as DST, was established by incorporating ligament footprint contour outlines onto the SSM of the bones. To this end, the SSMs of the femur and tibia were first deformed and sequentially best-registered to the 27 individual bone surface models (see “Subjects” section) by minimizing the sum of the squared distances between the SSM nodes and the vertices of the bone surface. Next, the vertices of the ligament footprint models were projected onto the nearest faces of the deformed SSM. The outer boundary of the projected footprint defined the footprint contour of the ligament (Fig. 1c), which was represented by 36 nodes and expressed in a barycentric coordinate system (BCS). Specifically, the coordinate of each node on the contour is considered as a linear combination of the corresponding polygonal vertices.

Following the establishment of individual ligament footprint contours, the Cartesian coordinates of contour nodes on the mean shape of the SSM were computed based on their respective barycentric coordinates. The footprint contours from all samples were averaged to obtain the mean contour (black outlines in Fig. 1d), which was expressed in BCS. The SSM of the bones, in combination with the averaged footprint contours, constituted the DST used for footprint contour estimation.

### Ligament footprint estimation

**Figure 2:**
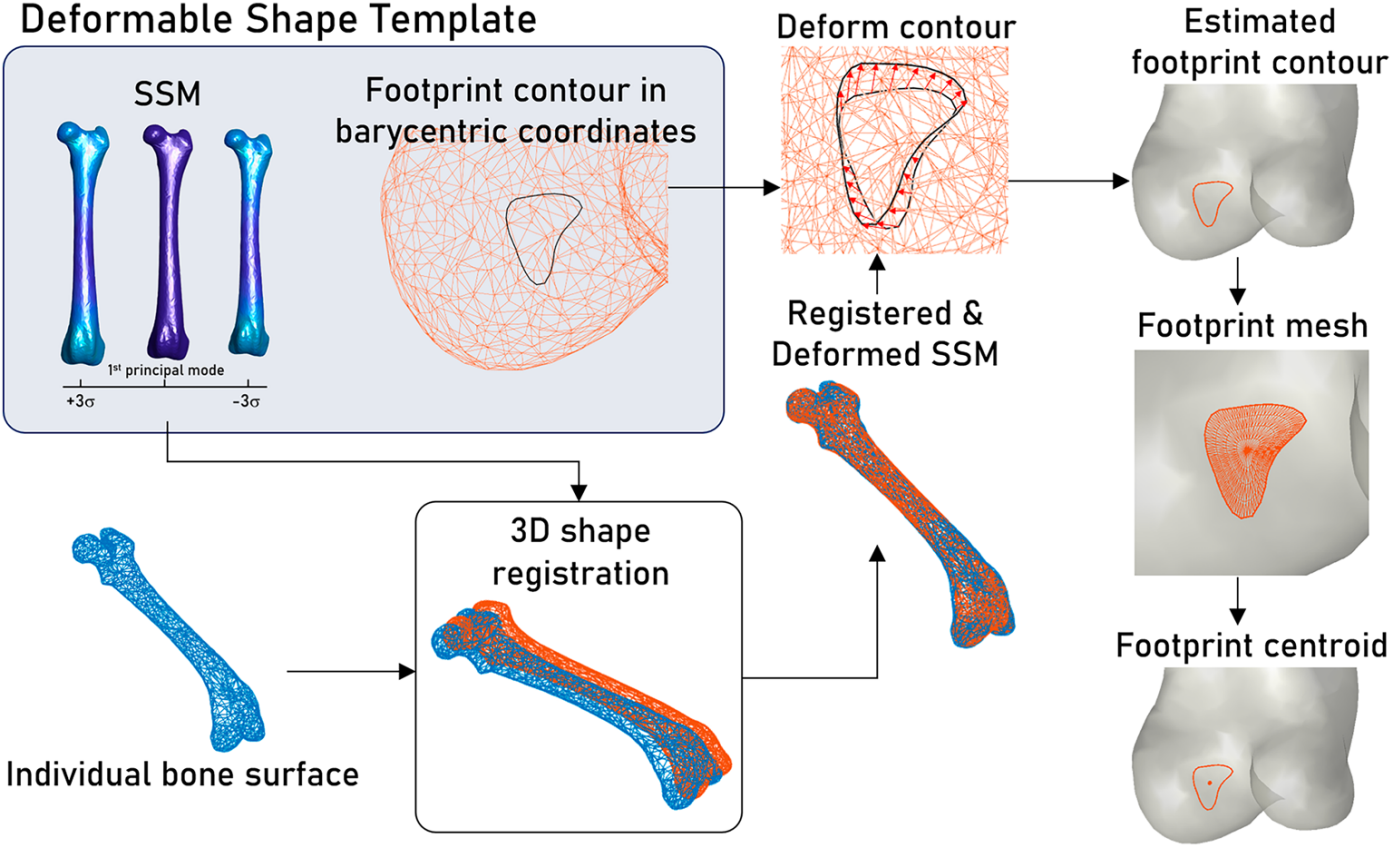
The step-by-step procedure for the estimation of individual ligament footprints.

The process of estimating the ligament footprint is outlined in Fig. 2. Given the DST of the target bone, the bone shape represented in the SSM within the DST can be customized to optimally match the point cloud of the individual bone shape. This customization is achieved by finding the optimal combination of pose and shape parameters of the SSM using the sequential quadratic programming method^22^ such that the sum of squared distances between the SSM nodes and the nearest points in the surface point cloud is minimized. The deformed SSM governs the positional and morphological modifications of the respective footprint contours within the DST. Within the customized footprint contour, a polygonal mesh is generated with all nodes adhering to the bone surface. The area and center of each polygon are computed. The centroid of the footprint is approximated by calculating the weighted average of all polygonal centers, where the weights are determined by the respective polygonal area. The total area of the footprint is obtained by summing the areas of all polygons. Note that, the number of polygonal meshes for each ligament footprint was set to 576 since a further subdivision of meshes yielded negligible changes in footprint centroid positions (< 10^–3^ mm). In the present study, the construction of both the SSM and DST, as well as the estimation of ligament footprints, were carried out using custom programs developed in MATLAB.

### Data analysis

The dispersion of the ligament footprint centroids over samples was analyzed on the mean shape of the SSM and was defined as the standard deviation (SD) of the coordinates of footprint centroids. The higher the SD value is, the greater the dispersion of the footprint centroids. The dispersion is related to interindividual variability and experimental errors, and the former is expected to be the major factor^11^.

The accuracies of the DST in estimating the footprint contours, centroids, and areas were analyzed using a leave-one-out cross-validation framework, where the target dogs were excluded during the DST generation process. The DST-estimated footprints were compared against the reference footprints obtained from CT-derived ligament footprint models (Fig. 1c).

The agreements in footprint centroids along three anatomical axes, namely cranial/caudal (Cr/Ca), proximal/distal (P/D), and lateral/medial (L/M), between DST-estimated and reference values were assessed, where the means (bias), SDs (precision), and root-mean-square (RMS) of differences and the 95% limit-of-agreement were calculated. The anatomical axes were defined according to a previous study^23^. A pair *t*-test was conducted to compare the estimated and reference footprint centroids.

The Euclidean distance between the reference and estimated footprint centroids was also calculated and referred to as the footprint centroid error. The footprint contour error was defined as the averaged Euclidean distances among 36 nodes between the estimated and reference footprint contours. The footprint area error was defined as the differences between the estimated and reference areas.

To compare the errors of the centroids, contours, and areas among different ligament footprints, Welch's one-way analysis of variance was performed due to unequal variances of data groups, as confirmed by Bartlett’s test. If significance was detected, the Dunnett T3 test was employed for post hoc analysis. The significance level was set at α = 0.05 for all statistical tests. All statistical analyses were performed using SPSS (ver. 25.0, International Business Machines Corporation (IBM), USA).

## Results

The averaged root-mean-squared residual shape differences between the deformed SSMs and individual bone surface models were 0.5 mm for the femur and 0.4 mm for the tibia. The results indicated that the deformed SSMs achieved accurate representations of individual bone shapes. It should be noted that the SSM of the fibula was not established in the present study, leading to the unavailability of the DST for estimating the distal footprint of the LCL.

**Figure 3:**
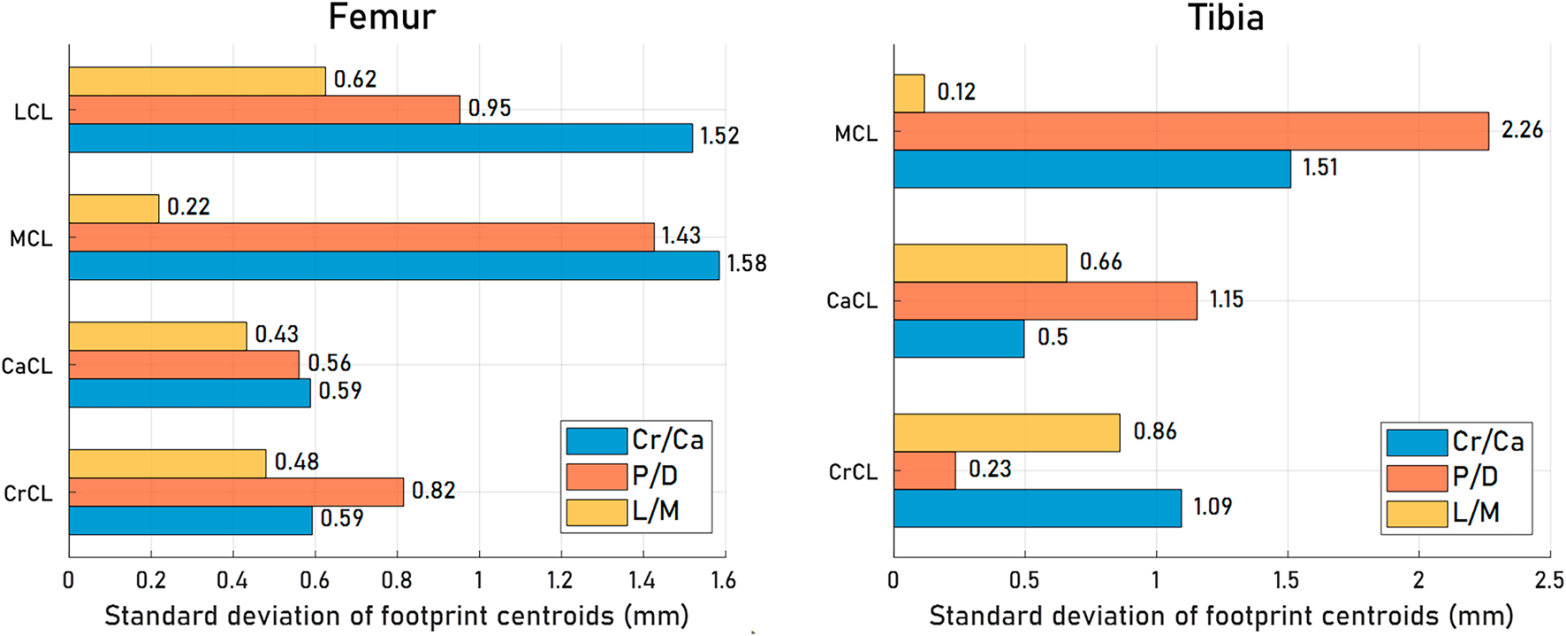
The dispersions of ligament footprint centroids. The dispersions of each ligament footprint centroid in the cranial/caudal (Cr/Ca), proximal/distal (P/D), and lateral/medial (L/M) directions were defined as the standard deviation of centroid coordinates across all subjects.

The dispersions of the cruciate ligaments (CrCL and CaCL) on the femur and tibia were less than 1.1 mm, 1.2 mm, and 0.9 mm in the cranial/caudal (Cr/Ca), proximal/distal (P/D), and lateral/medial (L/M) directions, respectively (Fig. 3). The corresponding values for collateral ligaments were 1.6 mm, 2.3 mm, and 0.6 mm, respectively (Fig. 3). Noteworthy, the footprint centroids of the collateral ligaments at the femoral site exhibited the most prominent divergence along the Cr/Ca direction, whereas the MCL footprint at the tibial site demonstrated the highest dispersion in the P/D direction.

**Figure 4:**
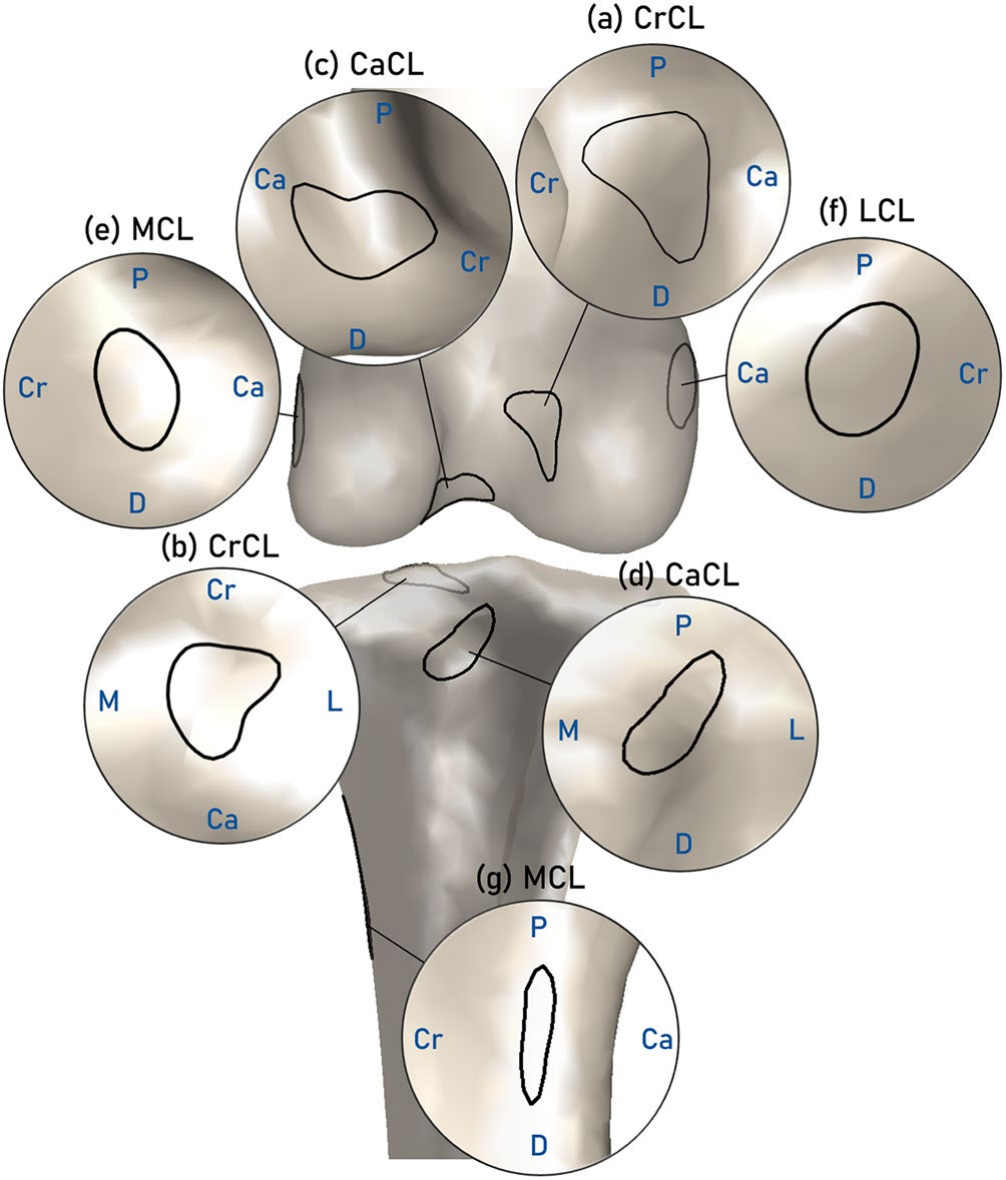
The shapes of stifle ligament footprints. The average shapes (black outline) of the (**a**) femoral CrCL footprint, (**b**) tibial CrCL footprint, (**c**) femoral CaCL footprint, (**d**) tibial CaCL footprint, (**e**) femoral MCL footprint, (**f**) femoral LCL footprint, and (**g**) tibial MCL footprint. Noted that the distal footprint of the LCL was not available.

Regarding the footprint shapes in our dataset, half of the femoral CrCL footprints display an asymmetrical arrowhead shape (14/27, 51.8%), while others exhibit shapes resembling rounded triangular (11/27, 40.7%) or ovate (2/27, 7.4%) forms. These variations collectively contribute to the formation of a rounded triangular pattern in the averaged contour (Fig. 4a). At the tibial site, the averaged CrCL footprint maintains a rounded triangular pattern, as the arrowhead shape of footprints was also observed in 14/27 (51.8%) individuals (Fig. 4b). Femoral CaCL footprints also show notable variability, with some resembling arrowheads (10/27, 37.0%), boomerangs (12/27, 44.4%), oblong-ovate (4/27, 14.8%) or circular shape (1/27, 3.7%), while the average contour appears as a bent oval with a subtle inward curvature on the proximal side (Fig. 4c). In contrast, the tibial CaCL footprints consistently exhibit an elongated elliptical shape (Fig. 4d). The collateral ligament footprints at the femoral site lack a consistent and distinct pattern, resulting in more circular averaged shapes (Fig. 4e, f). The tibial MCL attachment areas exhibit a distinctly elongated shape across all dogs observed, with an average footprint length-to-width ratio of approximately 5 (Fig. 4g).

The mean differences between the estimated and reference footprint centroids across three anatomical axes consistently fell within a 0.2 mm range, as indicated in Table 1. Notably, no statistically significant differences were observed in the coordinates of estimated and reference footprint centroids at any ligament attachment site (p > 0.05). The SDs of the estimated footprint centroid differences for cruciate ligaments were less than 1.0 mm in the Cr/Ca direction, 1.1 mm in the P/D direction, and 0.8 mm in the L/M direction, respectively. The corresponding values for the collateral ligaments were 1.4 mm, 2.1 mm, and 0.6 mm (Table 1). RMS differences were observed to be comparable to the SDs of the differences (Table 1). For the crucial ligaments, the 95% limit of agreement of footprint centroid differences ranged from − 1.4 to 1.4 mm at the femoral sites and from − 2.1 to 2.1 mm at the tibial attachment sites, both with the greatest values observed in the P/D component. The corresponding values for the collateral ligaments ranged from − 2.7 to 2.8 mm (femoral sites) and − 4.3 to 4.1 mm (tibial site, only MCL) as shown in Table 1.

**Table 1:** Comparison of footprint centroids between DST-estimated and reference values, presented as means ± standard deviations of differences, 95% limits of agreement, and root mean square (RMS) differences along the cranial/caudal (Cr/Ca), proximal/distal (P/D), and lateral/medial (L/M) directions. All values are expressed in millimeters. Noted that the DST for estimating the distal footprint of the LCL was not available.

	Ligament	Difference (mean ± SD)			95% limit-of-agreement [lower, upper]			RMS difference		
		Cr/Ca	P/D	L/M	Cr/Ca	P/D	L/M	Cr/Ca	P/D	L/M
Femur	CrCL	0.0 ± 0.6	0.0 ± 0.7	0.0 ± 0.5	[− 1.1, 1.1]	[− 1.4, 1.4]	[− 0.9, 0.9]	0.5	0.7	0.5
	CaCL	0.1 ± 0.5	0.0 ± 0.5	0.0 ± 0.4	[− 1.0, 1.1]	[− 1.0, 1.0]	[− 0.9, 0.8]	0.5	0.5	0.4
	MCL	0.1 ± 1.4	0.1 ± 1.3	− 0.1 ± 0.2	[− 2.7, 2.8]	[− 2.5, 2.6]	[− 0.5, 0.3]	1.4	1.3	0.2
	LCL	0.1 ± 1.4	0.0 ± 0.9	0.0 ± 0.6	[− 2.6, 2.8]	[− 1.8, 1.7]	[− 1.1, 1.1]	1.4	0.9	0.6
Tibia	CrCL	0.1 ± 1.0	0.0 ± 0.3	− 0.1 ± 0.8	[− 1.8, 1.9]	[− 0.5, 0.5]	[− 1.7, 1.5]	0.9	0.3	0.8
	CaCL	0.0 ± 0.6	− 0.1 ± 1.1	− 0.2 ± 0.6	[− 1.2, 1.1]	[− 2.1, 2.1]	[− 1.4, 1.1]	0.6	1.1	0.6
	MCL	0.0 ± 1.4	− 0.1 ± 2.1	0.1 ± 0.2	[− 2.7, 2.7]	[− 4.3, 4.1]	[− 0.4, 0.5]	1.4	2.1	0.2

**Figure 5:**
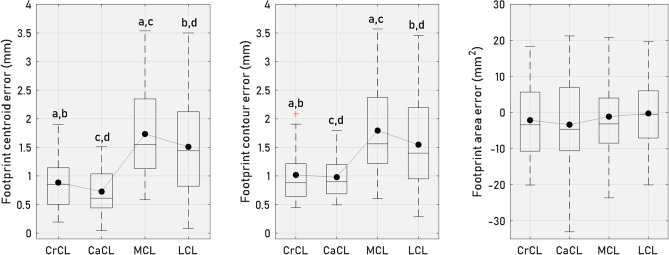
Boxplots of estimated ligament footprint errors at the femoral site. These boxplots display the distributions of centroid errors, contour errors, and area errors of the estimated footprints of the CrCL, CaCL, MCL, and LCL at the femoral site. The mean values are indicated by solid circle symbols, while the median values are represented by lines. The edges of the box delineate the 25th and 75th percentiles, with whiskers extending to encompass the most extreme values, excluding outliers. Groups with the same letters (a-d) differ significantly (*p* < 0.05).

**Figure 6:**
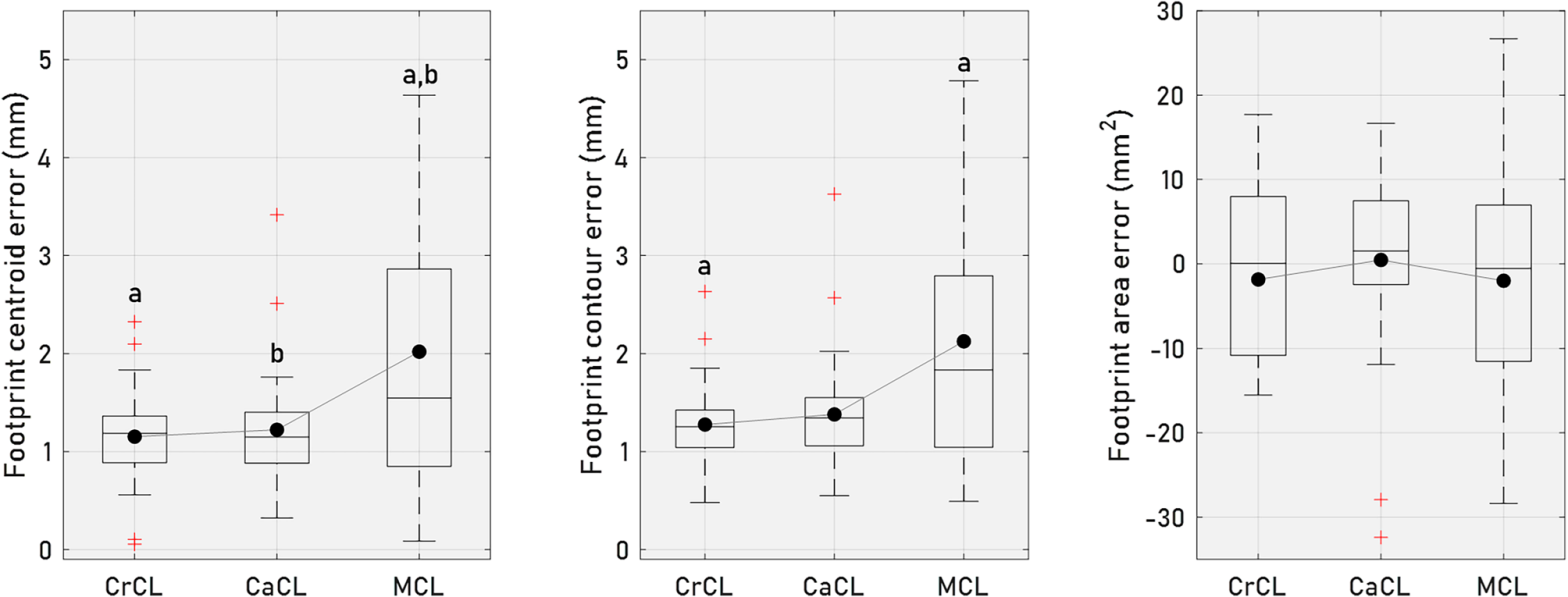
Boxplots of estimated ligament footprint errors at the tibial site. These boxplots display the distributions of centroid errors, contour errors, and area errors of the estimated footprints of the CrCL, CaCL, and MCL at the tibial site. The mean values are indicated by solid circle symbols, while the median values are represented by lines. The edges of the box delineate the 25th and 75th percentiles, with whiskers extending to encompass the most extreme values, excluding outliers. Groups with the same letters (a-b) differ significantly (*p* < 0.05).

The median values of the footprint centroid errors of the crucial ligaments were less than 0.9 mm at the femoral site and 1.2 mm at the tibial site. The median footprint centroid errors for the collateral ligaments were below 1.5 mm for both the femur and tibia. (Figs. 5 and 6). It was observed that the footprint centroid errors were significantly higher in collateral ligaments when compared to cruciate ligaments (Figs. 5 and 6). Furthermore, no significant differences were found in footprint centroid errors between CrCL and CaCL, or between MCL and LCL. Similar results were observed in footprint contour errors, with median errors for the crucial ligaments falling below 0.9 mm at the femoral site and 1.3 mm at the tibial site. The corresponding values for the collateral ligaments were 1.6 mm and 1.8 mm, respectively (Figs. 5 and 6). For the estimated footprint area, the median errors were within 4.6 mm^2^ across all ligament footprints; however, notable interdog variability was observed. At the femoral site (Fig. 5), footprint area errors ranged from − 38.4 to 21.2 mm^2^, and at the tibial site (Fig. 6), errors ranged from − 28.4 to 26.7 mm^2^.

## Discussion

In the present study, we first propose a prediction method to provide a 3D, noninvasive estimation of canine ligament footprint information based on an established dataset, where stifle ligament footprints marked with radio-opaque paint and individual bone shapes were recorded by CT images. The procedure for establishing the dataset in the study was inspired by the work of Pillet et al.^14^, who conducted a similar experimental approach to quantitatively extract the endpoints of human knee ligaments. In contrast to previous methodologies that focused on estimating footprint centroid positions^12,14,15^, we developed an SSM-based prediction method that directly estimates footprint contours based on individual bone shapes. This is achieved by propagating the changes in bone shapes to the deformation and positional adjustments of the ligament footprint contours. One advantage of our approach is that it allows for the derivation of footprint centroid and area size directly from the contour information, eliminating the need for additional models to achieve this objective.

Various methods have been proposed to quantify or mark ligament footprints, including using an optical tracking system^24^, identifying footprints in MR images^12^, and marking footprints with wires^15^ and radio-opaque panting^14^ before CT scanning. While the optical tracking system allows for accurate recording of footprint locations by precisely pointing to the target positions, extracting the complete bone shapes along with the footprint information can be challenging, particularly when the soft tissues are not entirely removed. As mentioned before, the direct identification of ligament footprints using any type of image data may be prone to variability among operators or even within the same operator^12^. In the present study, considering the small size of the cadaveric canine stifle used, the ligament footprints were marked with radio-opaque paint and underwent CT scans. This approach enabled the acquisition of high-resolution and high-contrast images, providing detailed visualization of both the ligament attachment areas and the bone shapes.

Obtaining patient-specific bone shapes is a prerequisite for estimating ligament footprints using DST. In this study, we employed CT to reconstruct the individual bone anatomy, thereby meeting the rigorous experimental requirements for achieving high accuracy and facilitating the identification of radio-opaque paints on the footprints. In addition, alternative options such as MR imaging and biplane X-ray reconstruction techniques^25^ are also viable for obtaining individual bone shapes. Nevertheless, it should be noted that the accuracy of the reconstructed bone shapes may vary depending on the imaging technique employed, and the determination of footprints relied on the deformed SSM best-fitting with individual bone shapes with the DST technique. Hence, further investigations are warranted to assess the implications of bone shape variations on the estimation of ligament footprints.

Based on the available footprint dataset, our observations indicated that half of femoral CrCL footprints manifest an arrowhead shape, aligning with previous reports that have documented the presence of this particular shape at the femoral CrCL footprints^15,26^. A similar shape was also observable at the tibial site (Fig. 4b). The femoral CaCL exhibited pronounced individual variations and, on average, presented a bent oval shape. The present study documented the footprint shapes of CaCL and tibial CrCL, which were not extensively explored in previous studies. However, due to the absence of comparative data available at this juncture, it remains inconclusive whether these shape characteristics are consistently present across various dog breeds.

The presence of noncircular shapes in the footprints reflects the two-bundle structure of the cruciate ligaments. Therefore, representing the ligament attachment area with a single point can only approximate the centroid of the two ligament bundle footprints. From the perspective of ligament reconstruction surgery, these predicted centroids can serve as a reference for drilling bone tunnels. In this study, the estimated centroid of the femoral CrCL footprint exhibited submillimeter RMS differences, with 95% limit-of-agreement values falling below 1.5 mm (Table 1). Considering the inherent challenge of clearly observing femoral CrCL footprint positions during canine CrCL intraarticular reconstruction surgery, the DST emerges as a tool that can be utilized in preoperative surgical planning and the development of surgical guidance.

Among the cruciate ligament footprints, the estimation error of the tibial CaCL footprint in the P/D direction was notably pronounced, with 95% limits of agreement slightly exceeding 2 mm (Table 1). This result could be linked to the increased positional variability observed in tibial CaCL footprints along the edge of the popliteal notch, where the dispersion value in the P/D component was also most pronounced (Fig. 3). This result is different from the observation in the human PCL endpoint analysis, wherein the prevailing aspect of endpoint dispersion was not attributed to the P/D component^14^. Similar observations can be found in the CrCL footprint at the tibial site, particularly in the Cr/Cd and L/M components, where the footprint centroid dispersion and estimation errors were concurrently higher (Fig. 3 and Table 1). The 95% limits of agreement values were found to slightly exceed 1.5 mm, as indicated in Table 1. Consequently, the averaged footprint contour on the DST and the resulting predicted centroids are less representative of individual scenarios at the tibial sites.

The collateral ligament footprints at the femoral site appeared to exhibit greater interdog variability in both shapes and positions (Fig. 3) compared to the crucial ligaments. Consequently, the averaged footprint shape proved to be less representative of the individual footprint shapes, affecting the DST-prediction outcome. Footprint centroid and contour errors at collateral ligaments were significantly greater than those at crucial ligaments (Fig. 5). The tibial MCL footprints also exhibited the most extensive dispersion in footprint centroids along the P/D direction among the stifle ligaments (Fig. 3), similar to observations in the human knee^14^. This poses challenges when attempting to accurately predict footprint locations with the DST. It is worth noting that the limited variability observed in the L/M components of the collateral footprint centroids can be attributed to the alignment of the normal directions of the bone surface around the collateral ligament attachment areas, which exhibited greater parallelism with the L/M axis of the bone.

Despite the findings of this study, it is essential to acknowledge certain limitations. First, the SSMs of the bones were created using bone shapes solely from healthy dogs and a limited number of dog breeds. This may limit the generalizability of the SSM to bones with degenerative changes and other dog breeds. Second, variations in ligament positions may exist among different breeds. The reliance on data solely from a specific breed in establishing ligament information could introduce errors when extrapolating to predict ligament footprints in other canine breeds. To enhance the generalization of the DST and better accommodate diverse skeletal and ligament morphological features across different canine breeds, considering the incorporation of advanced techniques (e.g., deep learning models) into DST may help bridge the conformational differences among dog breeds. Third, the limited cross-section and cylindrical symmetrical shape of the fibula posed challenges in accurately reconstructing its 3D shape, thereby hindering the creation of the SSM and its subsequent shape-matching process. Consequently, anatomical information pertaining to the distal footprint of the LCL was not attainable in this study.

In conclusion, we successfully established the DST incorporating footprint contours of the stifle ligaments for the femur and tibia. The application of shape transformation on the DST provides a feasible approach for estimating subject-specific ligament footprints. Our results indicate that among the four stifle ligaments, the footprint centroids of the cruciate ligaments exhibit less dispersion, and the DST enables more accurate footprint estimations compared to those of the collateral ligaments. In particular, submillimeter RMS differences were achieved in estimating the femoral CrCL and CaCL footprint centroids, which is advantageous for preoperative planning in the anatomical reconstruction of deficient ligaments when patient-specific bone shapes are available in clinical settings. Furthermore, quantitative identification of ligament attachment areas can assist in the customization of biomechanical models for the stifle joint, ultimately facilitating subject-specific biomechanical analysis of the stifle joint.

## Data Availability

The reported data and deformable shape templates created in this study are available from the corresponding author upon reasonable request.
